# Myocardial Infarction With Non-obstructive Coronary Arteries: A Clinical Conundrum

**DOI:** 10.7759/cureus.64135

**Published:** 2024-07-09

**Authors:** Elizabeth L Allison, Wayne-Andrew Palmer, Keston Rattan, Nitish Seenarine, Ezra Schrem, Robert Mills, Cristina A Mitre

**Affiliations:** 1 Department of Internal Medicine, State University of New York Downstate Medical Center, Brooklyn, USA; 2 Department of Cardiology, State University of New York Downstate Medical Center, Brooklyn, USA; 3 Department of Internal Medicine, Veterans Affairs New York Medical Center, Brooklyn, USA; 4 Department of Cardiology, Veterans Affairs New York Harbor Healthcare System, Brooklyn Campus, Brooklyn, USA

**Keywords:** coronary artery disease (cad), acute coronary syndrome, non-ischemic cardiac pathologies, cardiac risk factors and prevention, myocardial infarction with non-obstructive coronary arteries (minoca)

## Abstract

Myocardial infarction with non-obstructive coronary arteries (MINOCA) is defined by the presence of positive cardiac biomarkers with clinical evidence of infarction, the absence of significant coronary stenosis (≥50%) on angiography, and the lack of alternative diagnosis for the index presentation. MINOCA poses a diagnostic and therapeutic challenge due to the various pathophysiologic mechanisms underlying its presentation. Coronary artery plaque disruption is recognized as a crucial mechanism contributing to MINOCA. Plaque rupture and thrombus formation with subsequent myocardial ischemia may occur without significant luminal narrowing. A high index of suspicion is needed to make an early diagnosis. Here, a 68-year-old African American male patient presented with substernal chest pain, nonspecific ST segment changes on electrocardiogram, and elevation in cardiac biomarkers only one day after undergoing diagnostic cardiac catheterization that revealed non-obstructed coronary arteries. This case provides an example of MINOCA occurring secondary to suspected coronary artery plaque disruption in the setting of recent cardiac catheterization.

## Introduction

Myocardial infarction with non-obstructive coronary arteries (MINOCA) is increasingly being considered a common cause of myocardial infarction (MI) among patients presenting with acute coronary syndrome (ACS). The diagnosis of MINOCA is made in patients with acute myocardial infarction (AMI) as defined by the fourth universal definition of myocardial infarction [1] combined with non-obstructive coronary arteries on angiography (i.e., no coronary artery stenosis of ≥50% in any major epicardial vessel) and no specific alternate diagnosis for the clinical presentation [2]. The prevalence of MINOCA is reported to range from 5% to 15% depending on the population examined, with a predilection toward females and patients of African American, Maori, or Pacific race and Latinx ethnicity [2]. Despite this increasing prevalence, MINOCA continues to remain a clinical conundrum as its etiology and diagnosis warrant investigation via the multimodality imaging of the underlying causes to appropriately achieve patient-specific treatment [3,4].

## Case presentation

A 68-year-old African American male with multiple comorbidities including hypertension, non-insulin-dependent diabetes mellitus, hyperlipidemia, persistent atrial fibrillation, heart failure with reduced ejection fraction (HFrEF) of 25%-30% with an implantable cardiac defibrillator (ICD), and chronic angina on dual antianginal therapy presented to the emergency room after an episode of localized, constant, substernal chest pain. This pain was different than his usual chronic angina as it occurred at rest, lasting 30 minutes before it self-resolved. The patient had a history of anginal episodes that were reproduced with exertion, his exercise tolerance being limited to two blocks.

**Figure 1:**
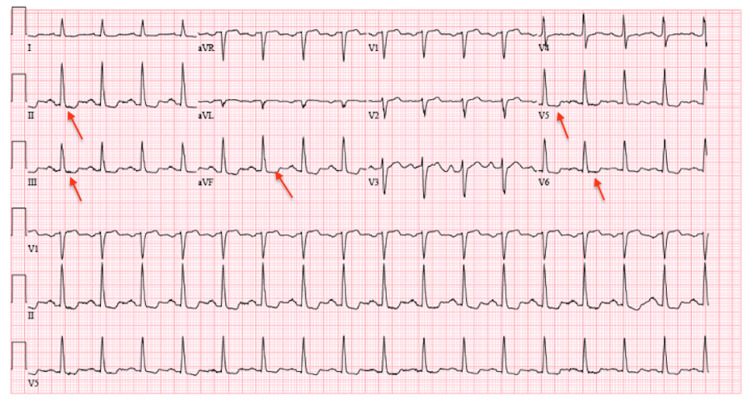
EKG on presentation. Sinus tachycardia with new ST segment/T wave changes in the inferior and lateral leads as indicated by the red arrows. EKG: electrocardiogram

**Figure 2:**
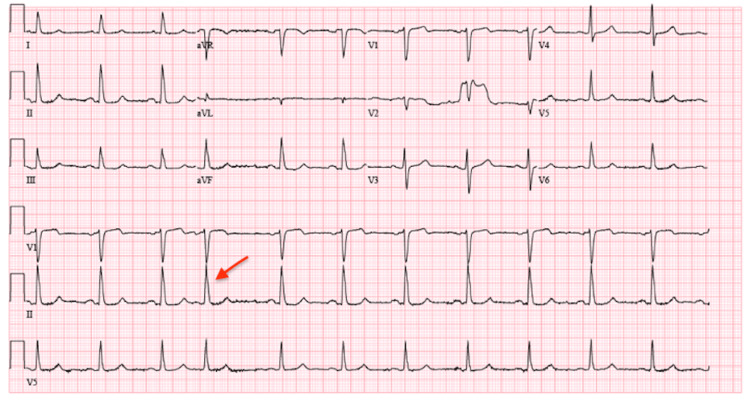
EKG one day prior. Sinus rhythm with premature atrial complexes (red arrow). EKG: electrocardiogram

**Figure 3:**
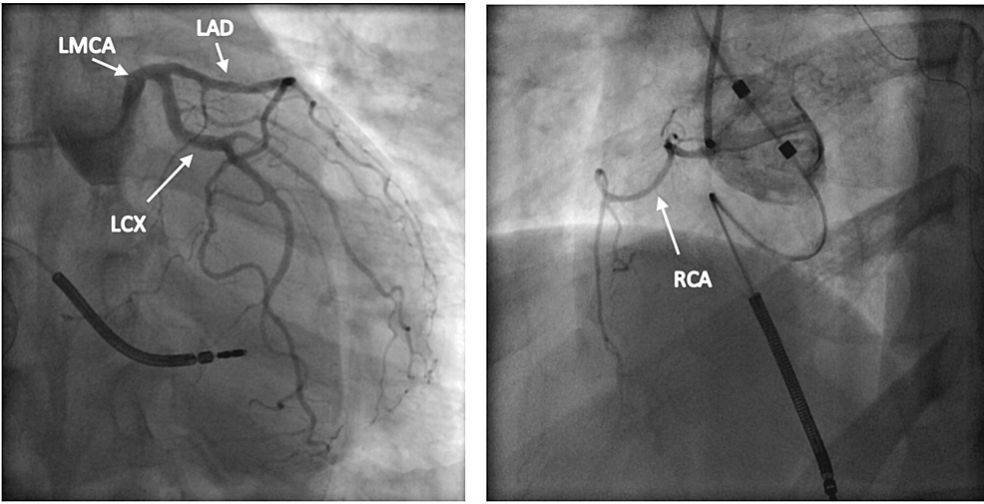
Non-obstructive CAD with 30% stenosis of the left main coronary artery (LMCA), 30%-40% of the left anterior descending artery (LAD), and 30%-40% of the left circumflex artery (LCX). Small non-dominant right coronary artery (RCA). CAD: coronary artery disease

Vital signs on initial presentation were significant for tachycardia and hypertension. Electrocardiogram (EKG) on presentation demonstrated sinus tachycardia with new ST/T changes suggestive of ischemia in the inferior and lateral leads (Figure 1) when compared to EKG performed the day before showing sinus rhythm with premature atrial complexes (Figure 2). Laboratory results were significant for an elevated high-sensitivity troponin of 47.8 ng/L, which subsequently increased to 143.3 ng/L, and an elevated creatinine of 1.5 mg/dL (Table 1). Previous workup for the patient’s chronic angina and cardiomyopathy included a left heart catheterization (LHC) performed one day prior to this presentation with chest pain and revealed non-obstructive coronary artery disease (CAD). Left heart catheterization demonstrated mild to moderate non-obstructive CAD with 30% stenosis of the left main coronary artery, 30%-40% of the left anterior descending artery, and 30%-40% of the left circumflex artery (Figure 3). The right coronary artery was small and non-dominant (Figure 3). Given the findings of non-obstructive CAD, no interventions were performed, and antianginal treatment was recommended.

**Table 1:** Laboratory values on admission. Hs-cTn: high-sensitivity cardiac troponin

Test name	Patient results	Reference range	Units
Hemoglobin	14.9	14.0-18.0	g/dL
White blood cell count	2.56	4.50-10.90	K/μL
Platelets	351	130-400	K/μL
Hs-cTn	47.8 to 143.3	≤14	ng/L
Potassium	4.4	3.5-5.2	mEq/L
Magnesium	1.5	1.5-2.4	mEq/L
Creatinine	1.50	0.70-1.20	mg/dL

A provisional diagnosis of MINOCA was made based on a rise and fall in high-sensitivity troponin with at least one value that was above the 99th percentile of the upper reference limit. Additionally, the patient had symptoms of myocardial ischemia, electrocardiographic findings suggestive of ischemia, and non-obstructed coronary arteries on diagnostic cardiac catheterization. Device interrogation did not show any significant arrhythmia. He remained asymptomatic while in hospital, troponin value decreased, EKG did not show any further changes, and he was discharged home. Repeat cardiac catheterization was not deemed necessary by cardiology. The patient was treated as per the secondary prevention guidelines for atherosclerosis as the cause for MINOCA could not be conclusively determined. He was seen in the cardiology clinic at a one-month follow-up post discharge when he was chest-pain-free with repeat EKG showing no evidence of ischemia.

## Discussion

The most common causes of acute coronary syndromes (ACS) are ST elevation myocardial infarction (STEMI) and non-ST elevation myocardial infarction (NSTEMI). High-sensitivity troponin is the most sensitive and specific marker of myocardial injury and ischemia and has a vital role in the diagnosis of ACS and MI. Troponin may be elevated without ACS when there is myocardial damage due to ischemia, nonischemic causes, or idiopathic myocardial injury.

In the presence of elevated cardiac markers, the diagnosis of MI includes at least one of the following: symptoms of myocardial ischemia, new ischemic changes on EKG, the development of pathological Q waves, imaging suggestive of ischemic etiology, and the identification of a thrombus by angiography or autopsy [5,6]. While most ACS occur due to underlying angiographically significant coronary stenosis with plaque disruption resulting in thrombosis and vessel occlusion, a smaller percentage occurs in the setting of non-obstructive CAD. This is known as MINOCA. The American Heart Association (AHA) defines MINOCA as acute MI fulfilling one of the following criteria: new ischemic changes on EKG, non-obstructive coronary arteries on angiography, and no specific alternate diagnosis for clinical presentation [2]. The underlying pathophysiologic mechanisms in MINOCA may include coronary artery plaque disruption that is not detectable by angiography, coronary vasospasm, coronary microvascular dysfunction, spontaneous coronary artery dissection (SCAD), and coronary embolism/thrombosis [7].

MINOCA is more common among female, African American, and Latinx patients under the age of 55 and accounts for 6% of MI cases overall. Diabetes, hypertension, hyperlipidemia, and smoking are well-established risk factors for cardiovascular morbidity and mortality. Similarly, these comorbidities are present in 75% of MINOCA cases [2]. Despite non-obstructive disease, MINOCA patients have a similar prognosis to obstructive CAD patients [8].

With anginal symptoms, significant elevations in troponin, and ST segment changes, this patient’s presentation was concerning for NSTEMI. Transient ST segment changes with troponin rise may be observed following cardiac catheterization; however, there was no stent deployment or angioplasty performed during the LHC. Therefore, while it’s of consideration, post-cardiac-catheterization troponin elevation and ST segment changes are less likely in this case. In the absence of an obvious stressor triggering elevations in troponin and ST changes, recent negative LHC, the resolution of symptoms, stable vitals, no significant arrhythmias on telemetry, and down-trending troponin value, a MINOCA diagnosis was considered, and conservative medical management was pursued. Studies show that 70%-80% of MINOCA patients present with NSTEMI [1]. It is possible that in this patient with a recent angiogram, MINOCA was due to coronary artery plaque disruption and less likely due to vasospasm or other causes such as arrhythmia as he denied any palpitations and had no events on telemetry. Myocarditis was unlikely as the patient had no signs or symptoms suggestive of an infection.

Nevertheless, a diagnosis of MINOCA is not solely made based on non-obstructive coronaries on coronary angiography. Additional imaging modalities including optical coherence tomography (OCT), intravascular ultrasound (IVUS), cardiac MRI (CMR), and cardiac CT (CCT) allow clinicians to further discern the etiology of a patient’s presentation with MINOCA [9]. MINOCA requires a thorough workup with a multimodality imaging approach to make the diagnosis and tailor appropriate treatment for patients.

This case reiterates the ways MINOCA may present and highlights the importance of the early identification of these patients as they have a similar prognosis to patients with obstructive CAD. The management of these patients requires good clinical judgment coupled with multimodality imaging, as there is no standard of care regarding the treatment of MINOCA [8].

## Conclusions

MINOCA remains a difficult diagnosis requiring noninvasive and invasive cardiac testing. Cardiac imaging modalities including IVUS, CCT, CMR, and OCT can provide useful insight to further evaluate the cardiac microvasculature and guide treatment accordingly. There is no standard of care regarding the management of MINOCA; as such, patients are treated using the best clinical judgment. The consensus recommendation is treatment targeting atherosclerosis or other identified underlying etiologies. Despite non-obstructive CAD, patients with MINOCA have a similar prognosis as patients with obstructive disease; therefore, early diagnosis and treatment are required to reduce major adverse cardiovascular outcomes.
